# Surface Impedance of Metasurfaces/Graphene Hybrid Structures

**DOI:** 10.1186/s11671-019-2995-x

**Published:** 2019-06-04

**Authors:** Han Xiong, Ming-Chun Tang, Yue-Hong Peng, Yuan-Hong Zhong, Xiao-Heng Tan

**Affiliations:** 1 School of Microelectronics and Communication Engineering, Chongqing University, Chongqing, 400044 China; 2 State Key Laboratory of Millimeter Waves, Southeast University, Nanjing, 211189 China; 3 School of Physics and Electronic Science, Chuxiong Normal University, Yunnan, 675000 China

**Keywords:** Surface impedance, Metasurface, Graphene

## Abstract

Understanding and manipulation of surface impedance in graphene hybrid structure is a significant issue for applications of graphene-based optoelectronics devices. In order to achieve this purpose in the terahertz region, analytical expressions for the impedances of metasurface were derived, which allows us to easily understand the relationship between physical dimensions and impedance. Simulation results show an excellent agreement with the analytical predictions. In addition, we focus on the synthetic impedance when square patch and graphene sheet joined together, discuss the influence of the size of metasurface as well as chemical potentiality as for graphene on the synthetic impedance. Based on these results, a number of absorbers as well as optical devices can be designed that utilize impedance metasurfaces.

## Introduction

In recent years, new artificial impedance metasurfaces, exhibiting anomalous electromagnetic properties, were proposed and investigated in the previous literatures [1–6]. Meanwhile, many kinds of metasurface applications have been introduced, such as holography [1], high-resolution imaging [2], carpet cloak [3], and absorbers [4, 5]. Metasurfaces can play a significant role in realizing the thin terahertz and optical devices. Nevertheless, due to the dispersive response by metasurfaces, many devices can only work in a single frequency band and the narrow spectrum cannot be tunable. Very recently, by varying the applied voltage at a broad range frequency such as terahertz or even optical frequencies, the conductivity can be controlled dynamically [7–10], that is why graphene proved that it is the best candidate for tuning the characteristics of plasmonic and metasurfaces structures [11]. Therefore, many devices designed by metasurface and graphene have been proposed [12–14].

In the meantime, several analytical models for calculating the equivalent impedance of metasurfaces or graphene sheet have been employed to explain the physical mechanism [8, 15–20]. Plane waves used for the excitation of graphene or metasurfaces models that can be divided into two different methods that are analytical and computational. Computational method is work on the Floquet expression [21, 22]. The advantage of using this method is that they are not restricted to the geometry of structures, and one of the most important merits is that it can provide accurate results. Nevertheless, commercial software using this method consumes considerable time and computational resources. On the other hand, a more precise and accurate analytical method is developed [23–27], it is easy to use and provide a better analysis of physical phenomena. In spite of the above-mentioned advantages, the challenges of achieving a high-precision analytical model for a specific metasurface unit are also prominent. Fortunately, considerable efforts and work have been made to predict the equivalent surface impedance and produced many excellent results [16, 28]. However, to the authors’ knowledge, the analytical model able to predict surface impedance of this hybrid combination is not yet known.

In this paper, a 3D artificial absorber was utilized to analyze and predict the impedance of metasurfaces/graphene hybrid structures, which takes into account the relationship between metasurfaces and graphene. For fast calculation of the surface impedance of metasurfaces, the analytical formulas were firstly developed. These simple and precise analytical formulas can allow a complete elucidation and basic requirement about impedance design. Then, the impedances of the graphene sheets are calculated. Finally, we focus on the relationship between the size of the metasurface, chemical potential *μ*_*c*_, and the impedance of the composite structure. Here, the surface impedance of metasurfaces/grapheme hybrid structure is discussed by calculating its real and imaginary components. To the best of our knowledge, there is almost no literature reported this mechanism comprehensively.

## Methods

### Impedances for Square Patches and Graphene Sheets

**Fig. 1:**
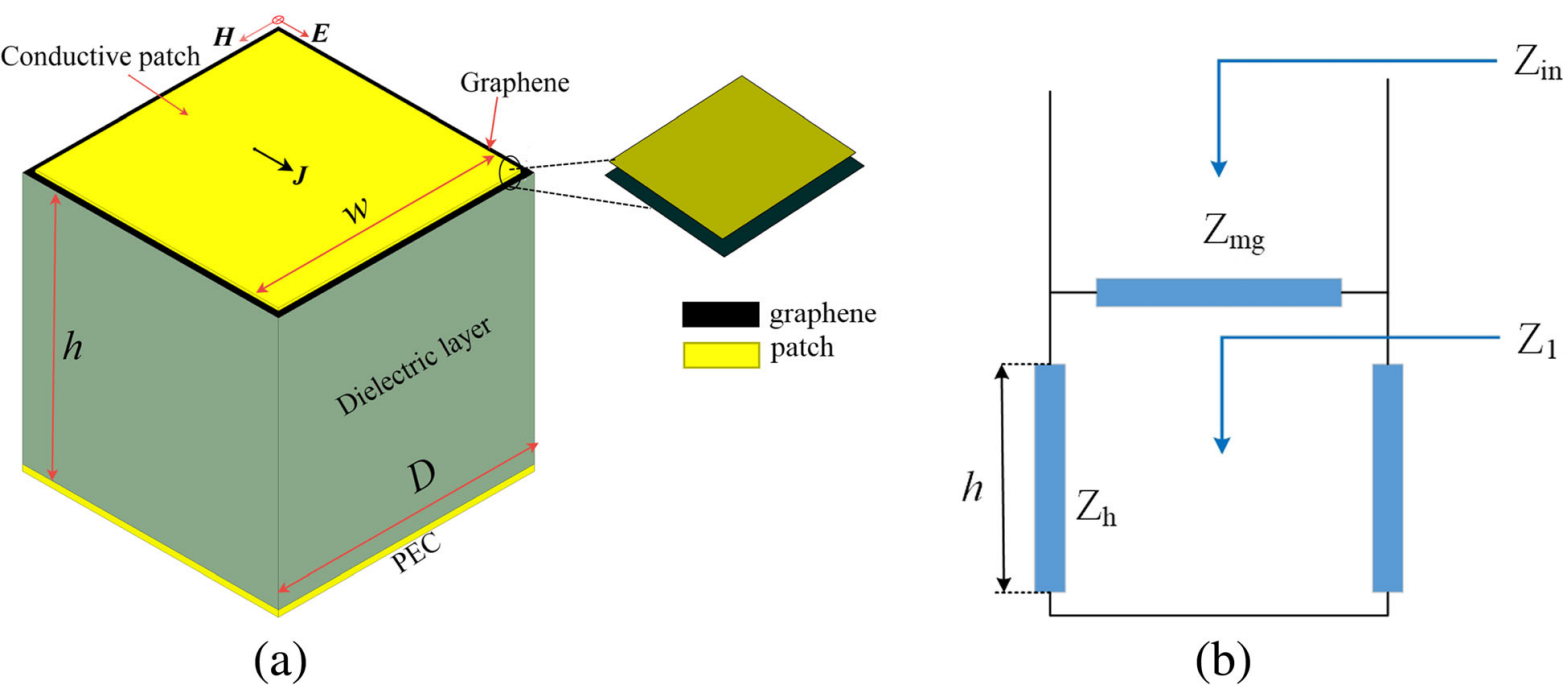
**a** Schematic of the metasurface-graphene absorber unit cell. **b** Local equivalent circuit model

A common structure of a metasurface-graphene absorber is presented in Fig. 1a. This simple structure absorber can be easily fabricated by surface micromachining. In this configuration, a thin conductive metasurface-graphene hybrid layer and the metallic ground plane are separated by a dielectric substrate as a spacer. The distance to the ground is *h*. For a small size square patch in comparison with the wavelength (period of array *D* ≪ *λ*) and patches are separated by a narrow slots (width of slot *D* − *w* ≪ *D*), the present model is valid. According to the transmission line theory, an equivalent circuit model of the absorptive structure can be constructed (shown in Fig. 1b), which can model the metasurface-graphene. A transmission line, short circuit, and the grid impedance *Z*_mg_, respectively, model the dielectric substrate section, ground plane, and the surface impedance of top patterned hybrid layered. According to the transmission line theory, the input impedance *Z*_in_ of this absorber can be established as follows:

1 $$ \frac{1}{Z_{in}}=\frac{1}{Z_1}+\frac{1}{Z_{mg}}=\frac{1}{j{Z}_h\ast \tan \left({k}_{zh}h\right)}+\frac{1}{Z_{mg}} $$

Where *Z*_h_ and *k*_zh_ are the impedance of the substrate layers and propagation constant in this region, respectively. Then, the absorptivity at the normal incidence can be calculated by

2 $$ A\left(\omega \right)=1-R\left(\omega \right)=1-{\left|{S}_{11}\right|}^2=1-{\left|\frac{Z_{in}-120\pi }{Z_{in}+120\pi}\right|}^2 $$

It is obvious that the impedance of metasurface-graphene sheet can be extracted from the simulated reflection coefficient. The relationship between the size of the conductive patch and the chemical potential *μ*_*c*_ can be found.

### Impedance for Square Patches

When the plane-wave is perpendicular to the metasurface, the array of planar patches acts as a capacitive grid (as shown in Fig. 1a). Surface impedance *Z*_m_ can be illustrated as the electromagnetic properties of square patches that relate the average current intensity 〈*J*〉 and the averaged electric field strength 〈*E*〉 in the plane of patch:

3 $$ \left\langle E\right\rangle ={Z}_m\left\langle J\right\rangle $$

In the case of a lossy pure resistive sheet impedance *Z*_s_ (im Z_s_=0), at normal incidence the equivalent impedance of the patch is represented by *Z*_m_, and can be expressed as follows [9, 18]:

4 $$ {Z}_m=\frac{D}{w}{Z}_s-j\frac{\eta_{eff}}{2\alpha } $$

Where ${\eta}_{\mathrm{eff}=}\sqrt{\mu_0/{\varepsilon}_0{\varepsilon}_{\mathrm{eff}}}$ represents the wave impedance of the uniform host medium, and *D*/*w* is the geometric element. The effective relative permittivity can be approximated as

5 $$ {\varepsilon}_{\mathrm{eff}}\approx \frac{\left({\varepsilon}_r+1\right)}{2} $$

Furthermore, the grid parameter *α* for an electrically dense array of ideally conducting patches can be written as

6 $$ \alpha =\frac{k_{\mathrm{eff}}D}{\pi}\ln \left(\frac{1}{\sin \frac{\pi w}{2D}}\right) $$

${k}_{\mathrm{eff}}={k}_0\sqrt{\varepsilon_{\mathrm{eff}}}$ is the wavenumber in the effective host medium. In free space, *μ*_0_, *ε*_0_, and *k*_0_ are the permeability, permittivity, and the wave number, respectively. Furthermore, it is worth to point out that relation (4) is valid when the wavelength *λ* is much greater than *D*.

According to the equation (2), we can find that the equivalent impedance is not only determined by the material sheet resistivity, but also by the array period *D* and width *w* of the structure parameters. To verify the certainty of such analytical formulas, the results obtained by full-wave simulations are presented and compared against the analytical solutions. The simulation discussed here was performed by using commercially available software Ansoft HFSS. For obtaining the reflection characteristics of the metasurface-graphene absorber unit cell, the periodic boundary conditions and Floquet ports were implemented. During its simulation, the pure resistive sheet impedance with *Z*_s_ = 35 Ω/sq is deposited on the substrate with thickness *h* = 20 μm, length *D* = 20 μm, and the relative permittivity of *ε*_*r*_ = 3.2(1 − *j*0.045). In order to extract the patch impedance *Z*_m_, according to the relationship between the simulated input impedance *Z*_in_ and the surface impedance of the grounded dielectric slab *Z*_*g*d_, the impedance of the metasurface patch can be expressed as follows:

7 $$ {Z}_m=\frac{Z_{in}{Z}_{gd}}{Z_{gd}-{Z}_{in}} $$

Where *Z*_*gd*_ = *jZ*_*d*_ tan(*k*_*d*_*h*), ${Z}_d=\sqrt{\mu_0/{\varepsilon}_0{\varepsilon}_r}$ is the characteristic impedance of the slab, ${k}_d=\omega \sqrt{\mu_0{\varepsilon}_0{\varepsilon}_r}$ is the propagation constant orthogonal to the surface of the substrate for the TEM mode.

**Fig. 2.**
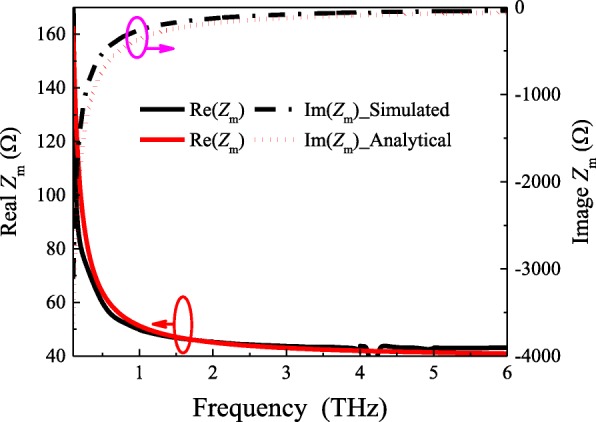
Simulated and analytical grid impedances of patch array with *w* = 19 μm

Analytical results are verified by comparison with the simulated ones based on the extracted reflection coefficient, as shown in Fig. 2. The black curves show the simulated results while the red curves are computed by using the proposed analytical expression. Although there exists a small difference between the simulated results and the theoretical predictions, this is due to Eq. (3) is an approximate equation. The overall trend is the same. Thus, confirming the validity and accuracy of our analytical expression for this model.

**Fig. 3:**
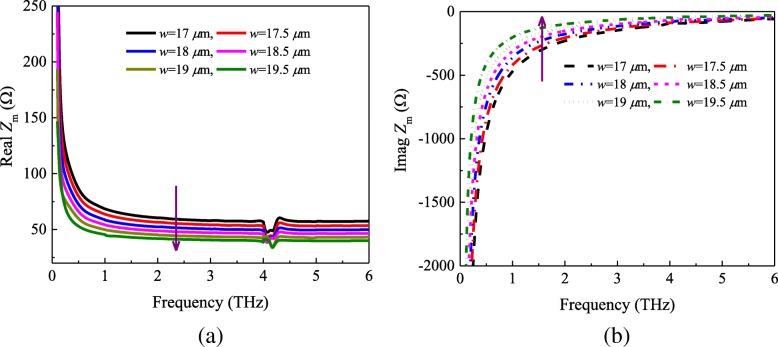
**a** Real and **b** imaginary part of the impedance *Z*_m_ with different sizes of the patch

In order to investigate the effect of the patch sizes on the impedance *Z*_m_ and validate the effectiveness of the formula (2), we performed the additional numerical simulation. Figure 3 plots the real and imaginary parts of the grid impedance *Z*_m_ for various geometrical parameters of the unit cell. From Fig. 3a, it can be observed that the real parts of the impedance *Z*_m_ decreases as the parameter *w* increases from 17 to 19.5 μm. According to Eq.2, we can find that the real parts of *Z*_m_ are inversely proportional to the patch length *w*. However, the imaginary parts show the opposite trend as shown in dotted lines (shown in Fig. 3b). Taking into account the Eqs. (2) and (3), the imaginary parts can be given by

8 $$ w\propto \ln \left(\mathit{\sin}\frac{\pi w}{2D}\right)\propto \frac{1}{\alpha}\propto \operatorname{Im}\left({Z}_m\right) $$

From the relation (8), we know that when *w* increases from 17 to 19.5 μm, the imaginary parts of the impedance *Z*_m_ will increase.

### Impedance for Graphene Sheets

Graphene can be seen as an infinitesimally thin surface. When there is no external magnetostatic bias and spatial dispersion, the surface conductivity *σ*_*g*_, can be computed by [29]

9 $$ {\sigma}_{\mathrm{g}}=\frac{j{e}^2{k}_BT}{\pi {\mathrm{\hslash}}^2\left(\omega +j/\tau \right)}\left[\frac{\mu_c}{k_BT}+2\ln \left({e}^{-{\mu}_c/{k}_BT}+1\right)\right]+\frac{j{e}^2}{4\pi \mathrm{\hslash}}\ln \left[\frac{2\left|{\mu}_c\right|-\left(\omega +j/\tau \right)\mathrm{\hslash}}{2\left|{\mu}_c\right|+\left(\omega +j/\tau \right)\mathrm{\hslash}}\right] $$

Where ℏ is the reduced Planck constant, *e* is the charge of an electron, *k*_B_ is the Boltzmann constant, while *μ*_c_, *ω*, *τ* and *T* are the chemical potential, angular frequency, relaxation time, and temperature, respectively. Here, we assume *T* = 300 K and *τ* = 0.1 ps throughout this study. The sheet impedance of graphene can be calculated as

10 $$ {Z}_g\left({\mu}_c\right)=1/{\sigma}_g={R}_g\left({\mu}_c\right)+j{X}_g\left({\mu}_c\right) $$

Where *R*_g_ and *X*_g_ are the surface resistance and reactance.

**Fig. 4:**
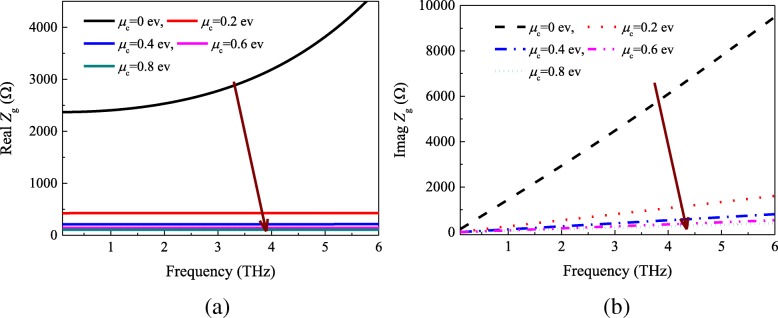
**a** Real and **b** imaginary parts of the surface impedance as function of frequency and chemical potential

The sheet impedance of graphene is calculated according to Eqs. (9) and (10). Figure 4 indicates the real and imaginary components of the surface impedance versus chemical *μ*_c_. We can find that the surface resistance and reactance continuously decrease with increasing *μ*_c_. Moreover, the real parts of the graphene sheet surface resistance keep almost unchanged in the range of 0.2−6 THz when the chemical potential is fixed at a certain value.

## Results and Discussion

**Fig. 5:**
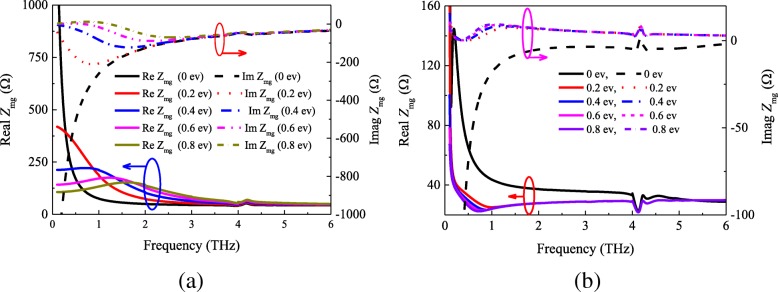
Metasurface-graphene film impedance *Z*_mg_ with different chemical potentials. **a** Analytical and **b** simulated results

In the case of a square patch on a graphene sheet, the surface impedance for this hybrid structure should be determined. In the prior literatures [8, 30–37], the total impedance at the surface of this hybrid structure *Z*_mg_ is equal to the parallel combination of the square patch impedance *Z*_m_ and the graphene sheet impedance *Z*_g_, i.e., *Z*_*mg*_ = *Z*_*m*_ ∥ *Z*_*g*_. However, through our simulation and calculation, it is found that this relationship is not valid. In order to verify the authenticity, we simulated a metasurface-graphene absorber unit shown in Fig. 1a, then retrieved the surface impedance of the film according to the Eq (1). Figure 5 shows the analytical and simulated values of the real and imaginary part of *Z*_mg_ at different chemical potentials with *w* = 19 μm.

From Fig. 5a, b, one can see that there exists great differences between the analytical and simulated results. Figure 5a shows that the real part of the analytical results was mainly concentrated between 40 and 500 Ω, and the imaginary part of the effective impedance ranges from − 210 to 0 Ω. Nevertheless, according to Fig. 5b, we can find that the values of the real part of impedance from 20 to 140 Ω, and the imaginary part is close to 0 by increasing *μ*_c_ from 0 to 0.8 ev. However, the analytical and simulated results show the same trend that the impedance will tend to be stable with increasing frequency. The reason is that the impedances of the graphene sheet and square patch become smaller when the frequency increases. It is noteworthy that, compared the impedance of metasurface-graphene film at 0 ev with the other results, the impedance *Z*_mg_ is quite different. This is due to the values of the graphene sheet impedance at 0 ev is quite different from the higher chemical potential (seen in Fig. 4).

**Fig. 6:**
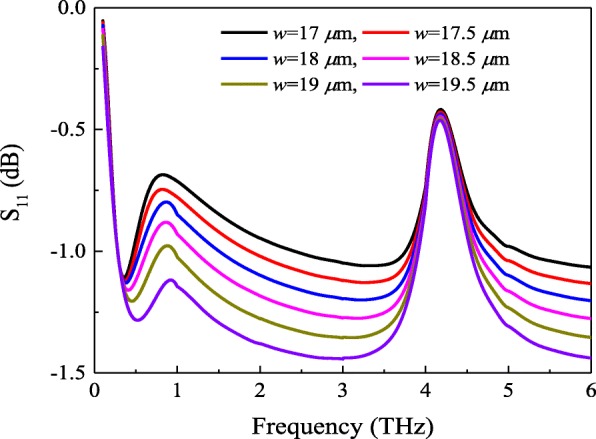
Reflection coefficient of the metasurface-graphene absorber with the chemical potential *μ*_c_ = 0.4 ev

**Fig. 7:**
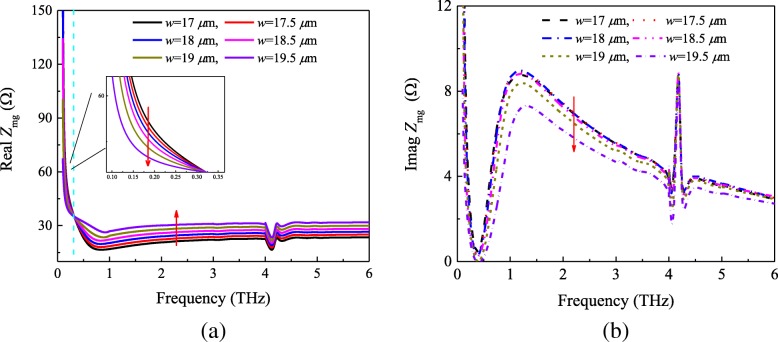
Metasurface-graphene film impedance *Z*_mg_ retrieved from *S*-parameters with the chemical potential *μ*_c_ = 0.4 ev. **a** Real and **b** imaginary parts

Thus, we can make the following conclusions from the calculated and simulated impedance in Fig. 5. First, the surface impedance of the metasurface-graphene film *Z*_mg_ is not strictly equal to the parallel combination of *Z*_m_ and *Z*_g_. However, second, there exists a certain relationship between them. In order to demonstrate these conclusions, we first simulate the structure of the absorber shown in Fig. 1 with varied patch sizes. The reflection coefficient of the metasurface-graphene absorber with the chemical potential *μ*_c_ = 0.4 ev is displayed in Fig. 6. According to the transmission line theory and model, the impedance *Z*_mg_ can be obtained. Figure 7 shows the real and imaginary components of the retrieved impedance *Z*_mg_ with different patch sizes. According to Fig. 7a, one can see that the real part of the metasurface-graphene film decreases in the beginning as the patch length *w* increases from 17 μm to 19.5 μm. However, the opposite trend is found when the frequency is higher than 0.31 THz. On the other hand, Fig. 7b indicates that the trend of the imaginary part is the same as the first half of Fig. 7a. Furthermore, comparing Figs. 4 and 5a, we found that there was a similar situation in Figs. 3 and 7. It also directly proves the above conclusions.

**Fig. 8.**
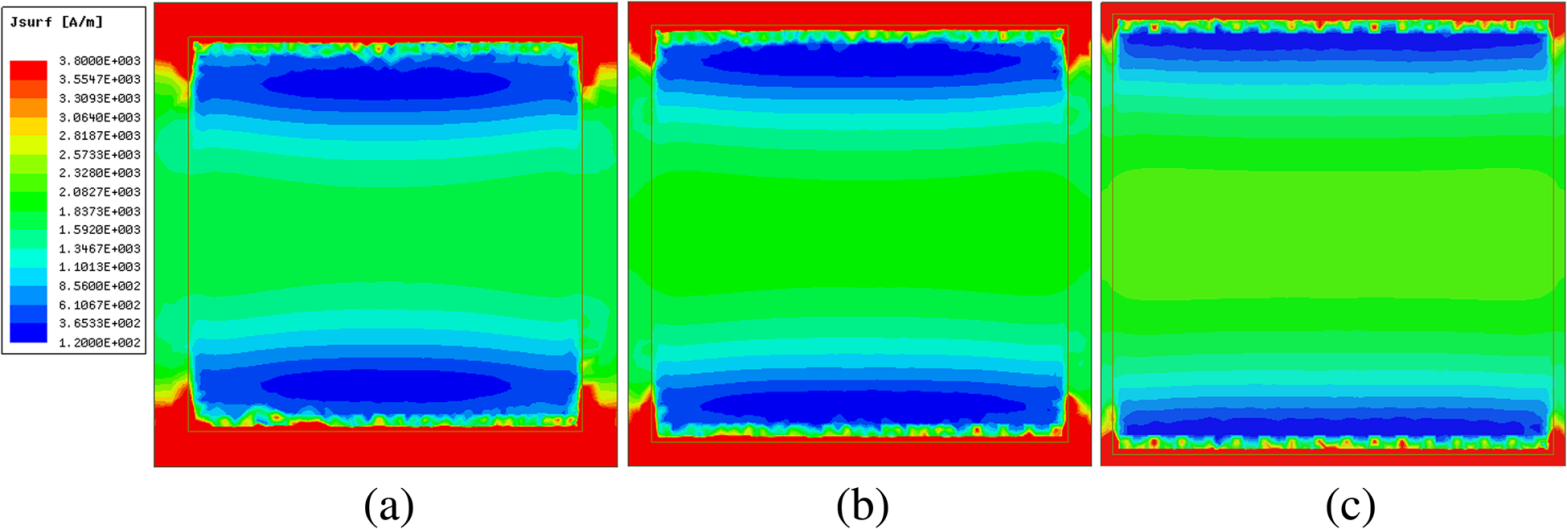
Magnitude of surface current with different patch sizes but at the same frequency. **a** *w* = 17 μm, **b** *w* = 18 μm, and **c** *w* = 19 μm

To further explore the physical origins of surface resistance as a function of patch size, the surface current distributions of the metasurface-graphene film in normal incidence are investigated at 3 THz. Figure 8 shows the variation in current intensity for *w* = 17, 18, and 19 μm with the chemical potential *μ*_c_ = 0.4ev. The color represents the intensity of the field. Obviously, as the size increases, the magnitude of surface current decreases. Taking into account Eq. 3 and Fig. 7a, when the electric field intensity is a fixed value at 3 THz, the film impedance of metasurface-graphene can be given by

11 $$ {Z}_{mg}\propto w\propto \frac{1}{J}\kern0.5em \left(f>0.32\ \mathrm{THz}\right) $$

From relation (11), we can find that the length of the patch is inversely proportional to the magnitude of surface current *J*. The qualitative agreement between the simulated and the theoretical results can be clearly observed. To quantificationally analyze this physical phenomenon, the integral value of the surface current distribution on the metasurface-graphene film is calculated by using the HFSS Fields Calculator, and the values are 1.10e-6, 1.07e-6, and 1.04e-6 A, respectively. These results are consistent with Fig. 8.

## Conclusions

In summary, for metasurface-graphene thin film in THz frequency, the fundamental and effective surface impedances were investigated. Analytical formulas were derived and verified for calculating the impedance of a square patch. As for the metasurface-graphene hybrid structure, the simulated results based on the extracted reflection coefficient were compared with the analytical results obtained from the parallel combination of the square patch and the graphene sheet impedances. Additional analysis was performed on discussing the effect of patch size on effective impedance. Furthermore, the relationships between patch size and film impedance were qualitatively and quantificationally explained by plotting and integrated the surface current. This analysis method can be extended to study the impedance problem with two other different conductive layers. In addition, extensive numerical simulation as well as analytically optimize composite layers for specially applied to antenna and absorber can be avoided by our analysis that made in this work.

## Abbreviations

HFSS: High-frequency structure simulation
TEM: Transverse electromagnetic
THz: Terahertz
